# Acoustic Mist Ionization Mass Spectrometry for Ultrahigh-Throughput Metabolomics Screening

**DOI:** 10.1021/acs.analchem.1c01616

**Published:** 2021-06-22

**Authors:** Matthew J. Smith, Delyan P. Ivanov, Ralf J. M. Weber, Jonathan Wingfield, Mark R. Viant

**Affiliations:** † School of Biosciences, University of Birmingham, Edgbaston, Birmingham B15 2TT, U.K.; ‡ Mechanistic Biology & Profiling, Discovery Sciences, R&D, AstraZeneca, Cambridge CB4 0WG, U.K.

## Abstract

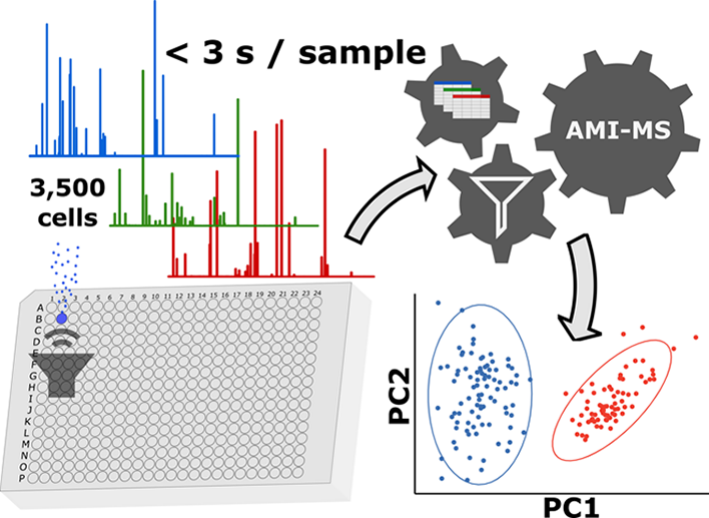

Incorporating safety data early in the drug discovery pipeline is key to reducing costly lead candidate failures. For a single drug development project, we estimate that several thousand samples per day require screening (<10 s per acquisition). While chromatography-based metabolomics has proven value at predicting toxicity from metabolic biomarker profiles, it lacks sufficiently high sample throughput. Acoustic mist ionization mass spectrometry (AMI–MS) is an atmospheric pressure ionization approach that can measure metabolites directly from 384-well plates with unparalleled speed. We sought to implement a signal processing and data analysis workflow to produce high-quality AMI–MS metabolomics data and to demonstrate its application to drug safety screening. An existing direct infusion mass spectrometry workflow was adapted, extended, optimized, and tested, utilizing three AMI–MS data sets acquired from technical and biological replicates of metabolite standards and HepG2 cell lysates and a toxicity study. Driven by criteria to minimize variance and maximize feature counts, an algorithm to extract the pulsed scan data was designed; parameters for signal-to-noise-ratio, replicate filter, sample filter, missing value filter, and RSD filter were all optimized; normalization and batch correction strategies were adapted; and cell phenotype filtering was implemented to exclude high cytotoxicity samples. The workflow was demonstrated using a highly replicated HepG2 toxicity data set, comprising 2772 samples from exposures to 16 drugs across 9 concentrations and generated in under 5 h, revealing metabolic phenotypes and individual metabolite changes that characterize specific modes of action. This AMI–MS workflow opens the door to ultrahigh-throughput metabolomics screening, increasing the rate of sample analysis by approximately 2 orders of magnitude.

## Introduction

Safety concerns are the major cause of drug candidate failures in both preclinical and Phase 1 trials.^1^ The high costs of failures at such a late stage of development have a significant impact on pharmaceutical productivity,^2^ and therefore, reducing the safety liability remains a priority. Being able to incorporate high-value on- and off-target safety data into decision making, early in the drug discovery pipeline, is key to reducing this attrition. One approach to safety screening is to measure a defined panel of molecular key event biomarkers that can predict specific modes of action (MoA’s) and/or adverse outcomes (AOs).^3−5^ While several metabolic biomarkers of toxicity are known,^6^ the optimal composition of a metabolic biomarker panel that can predict multiple MoA’s has yet to be discovered. Instead, omics technologies are attempting to address this knowledge gap, particularly via their ability to detect off-target toxicities. Metabolomics provides the most downstream molecular phenotype of the omics approaches and has been used to identify early response biomarkers that are indicative of AOs. A significant bottleneck, however, is the relatively low sample throughput of metabolomics analyses, typically requiring chromatographic separation and mass spectrometric detection to analyze ca. 100 samples per day, per mass spectrometer.^7^ In contrast, for a single drug development project, we estimate the need to analyze several thousands of samples per day, equivalent to substantially less than 10 s per metabolomics analysis. If achievable, ultrahigh-throughput (UHT) metabolomics may enable a step change in our ability to generate high-impact safety data for hundreds of drug candidates per week, early in development, and may also provide vital mechanistic insights into the pathways involved in toxicity.

Several analytical systems are available for high-throughput metabolomics, which can screen hundreds of putatively annotated metabolites, although these systems are not intended to definitively identify metabolites nor to fully characterize cellular or biofluid metabolomes. For example, nanoelectrospray ionization direct infusion mass spectrometry (nESI–DIMS) offers a relatively rapid analysis in untargeted metabolomics studies (typically 1–3 min per sample) with high analytical sensitivity^8,9^ and has been utilized extensively in toxicology.^9,10^ Other systems such as the Agilent RapidFire offer improved throughput of 7 s per sample for enzyme screening^11,12^ and <1 min per sample for untargeted metabolomics^13^ but are yet to be widely adopted in toxicology. Surface analysis mass spectrometry approaches such as matrix-assisted laser desorption ionization (MALDI) and desorption electrospray ionization (DESI) can sample at rates of ca. 1 Hz, directly from extracts spotted onto microarrays for mass spectrometry.^14^ Such approaches have been applied to single-cell metabolomics;^15,16^ however, these techniques suffer increased sample preparation time associated with matrix application (MALDI only) as well as suppression of the small-molecule *m/z* range, caused by the applied matrix^17^ and solvent clusters in MALDI and DESI, respectively.

Acoustic mist ionization mass spectrometry (AMI–MS) has proven capable of measuring the deacetylation of acetylated human histone deacetylase (HDAC), in the presence of a library of HDAC inhibitors and HDAC enzyme, at a rate of 100,000 samples/day (anticipated limit of 3 samples/s)^3^ from 3500 cells/sample with just 0.002% carryover.^4^ More recently, AMI–MS has been shown to simultaneously detect many biologically relevant metabolites to infer MoA’s in treated MCF7 cell extracts with comparable throughput and sensitivity as the targeted assays.^18^

The atmospheric pressure ionization technique achieves this using an echo acoustic transducer and charging cone to generate a mist of nanoliter-sized charged droplets that are guided through an ion transfer line to a mass spectrometer.^3,4^ Application of AMI–MS to metabolomics could open a door to UHT screening, with applications in several fields, including toxicology.

To deploy AMI–MS metabolomics in a drug safety screening program, specifically to adapt it to the principles and experimental designs for both high-throughput screening and metabolomics, it requires extensive optimization of the data generation, signal processing, and analysis. The overall aim of this study was to implement a signal processing and data analysis workflow to ensure that high-quality AMI–MS data can be produced and to demonstrate its application to a UHT metabolomics screening study in drug toxicology. Specifically, the first objective was to deploy and optimize a multistep signal processing workflow that addressed the following challenges: (i) defining on- and off-scans in the polarity switching cycle of AMI–MS in order to extract meaningful real signals, (ii) filtering relatively low intensity, sparse, but reliable signals of the intracellular origin from noise, (iii) addressing intensity corrections through batch correction between sample plates and normalization across all samples, and (iv) processing the metabolic phenotypes of the samples consistently, even though they can range from healthy controls to completely cytotoxic, via parallel fluorescence imaging measurements and “cell phenotype filtering”. The second objective was to highlight the novel application of UHT AMI–MS metabolomics to toxicity screening through the rapid collection of HegG2 metabolic phenotypes in a highly replicated exposure study with 16 drugs each at 9 concentrations, using a 384-well plate format throughout exposures, sample preparation, and data generation.

## Experimental Section

### Preparation of Metabolite Standard and HepG2 Samples

Four AMI–MS metabolomics data sets were generated from either standards or extracts of HepG2 C3A cells to address each of the objectives described above, all prepared in 384-well plates. The sample sets are referred to throughout this paper as “standards”, “technical replicates”, “biological controls”, and “toxicity study” samples (see Supporting Information for further information). The samples of standards each contained a mixture of eight stable isotope-labeled and eight unlabelled metabolite standards in 50 μL of 25:75 (v/v) methanol/water (Table S1, Supporting Information). The technical replicates and biological controls comprised extracts of HepG2 cells that had been cultured for 48 h in bulk and then aliquoted across eight 384-well analysis plates or cultured in individual wells (seeded at 3500 cells/well) across three plates, respectively, washed and then lysed with methanol and aliquoted into analysis plates (see Supporting Information). The toxicity study samples were derived as for the biological controls; negative control samples [dimethyl sulfoxide (DMSO)-controls] remained untreated, but the remaining samples were additionally treated for 24 h with each of the 16 drugs (Table S2) at nine half-log concentrations from 31.6 nM to 316 μM, and the positive control samples (death-controls) were formed through treatment with 316 μM chlorpromazine.

The study included *n* = 18 biological replicates per treatment group resulting in a total of 2772 samples, distributed across nine 384-well plates, as previously described.^19^ Every sample for AMI–MS analysis, from all four sample sets, contained 6 of the stable isotope-labeled standards for use as a calibrant (Table S1) and were made up to a final volume of 50 μL of 25:75 (v/v) methanol/water. “Cell imaging” data were generated for the toxicity study by staining the cellular DNA bound to the culturing plates following cell lysis, with Hoechst 33342 and analyzing using a fluorescence microscope CellInsight (Thermo Fisher Scientific). This cell count was used as a proxy for the number of live cells in the sample prior to cell lysis, as the majority of apoptotic and dead cells were removed by the washing steps (see Supporting Information).

### AMI–MS Data Acquisition

AMI–MS instrument configuration and methods were as previously described by Sinclair et al.^4^ In brief, the acoustic wave was provided using an Echo 555 liquid handler (Labcyte Inc., Sunnyvale, CA) positioned beneath an XY-stage (Labcyte custom-made) holding the 384-well plate. A charge cone with a high-voltage power supply (RIGOL) and a heated transfer tube (Waters, Wilmslow, UK—custom-made) held above the plate formed and guided the ions into the Xevo G2-XS quadrupole time-of-flight mass spectrometer (Waters). The ionization process inferred particular demands on the signal processing. Specifically, the charging cone (+3 kV) placed above each test well (in the 384-well plate) induced an accumulation of negative charge on the liquid surface. Each time an acoustic wave was pulsed through the sample (at 1400 Hz), a mist of nL-sized droplets, carrying the negative charge, was generated. To discharge the well and circumvent charge build up, the charging cone polarity was inverted after every 10 nL package of sample dispensed. From the total of 100 nL dispensed per sample, five packages of negative ions were measured at the mass detector, which we refer to as “on-scan” data. Typically, the first package of droplets from each sample was fired using the “off” polarity (an “off-scan”) to settle the meniscus; hence, the desired signals were from the second, fourth, and so forth ion packages. The droplet mist was drawn into a heated capillary (250 °C), which along with a nitrogen cone gas flow of 50 L/h, aided desolvation of the sample as it transited into the ion source (100 °C). The mass detector scanned for 80 ms with an interscan delay of 14 ms across a mass range from 50 to 1200 Da. This enabled an average of 3 scans per 10 nL ion package (and polarity switch), hence a total of 15 on-scans per sample.

### AMI–MS Data Processing and Analysis

**Figure 1:**
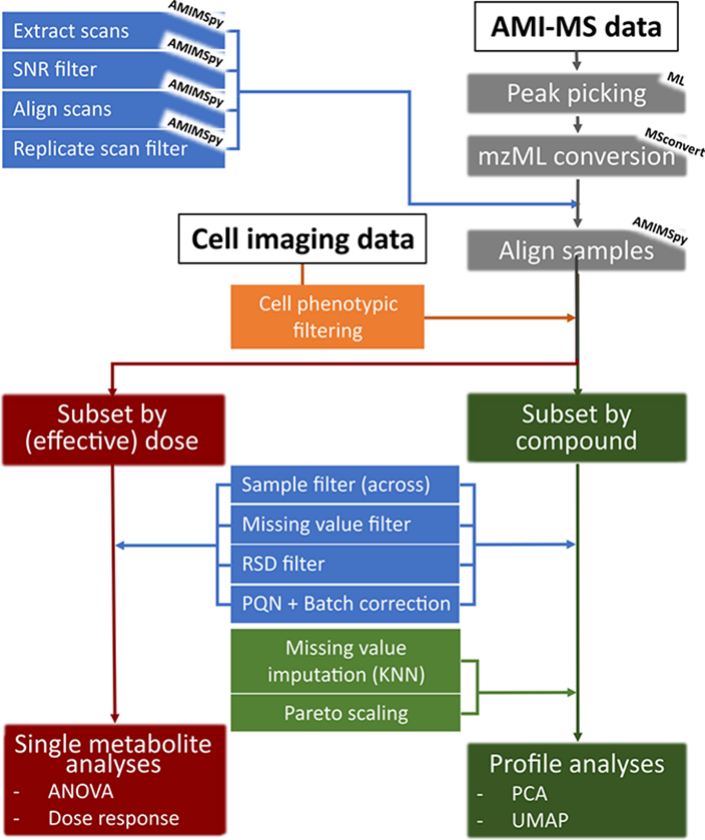
Data-processing workflow from raw AMI–MS and cell imaging data to single metabolite and profile analyses. The “ML” (MassLynx) MSconvert and “AMIMSpy” labels pertain to the required software for these processing steps.

Implementing and optimizing a data-processing workflow involved sequentially considering each of the components outlined in Figure 1, using the appropriate standards and/or HepG2 extract data sets. The raw AMI–MS data were converted to .mzML format using MSConvert (freeware, http://proteowizard.sourceforge.net/) before being processed using AMIMSpy (https://github.com/computational-metabolomics/amimspy), a new software package that has been derived from DIMSpy (https://github.com/computational-metabolomics/dimspy), the latter being developed over the last decade specifically to process direct infusion mass spectrometry metabolomics data.^8^

### Extracting Raw Scan Data

**Figure 2:**
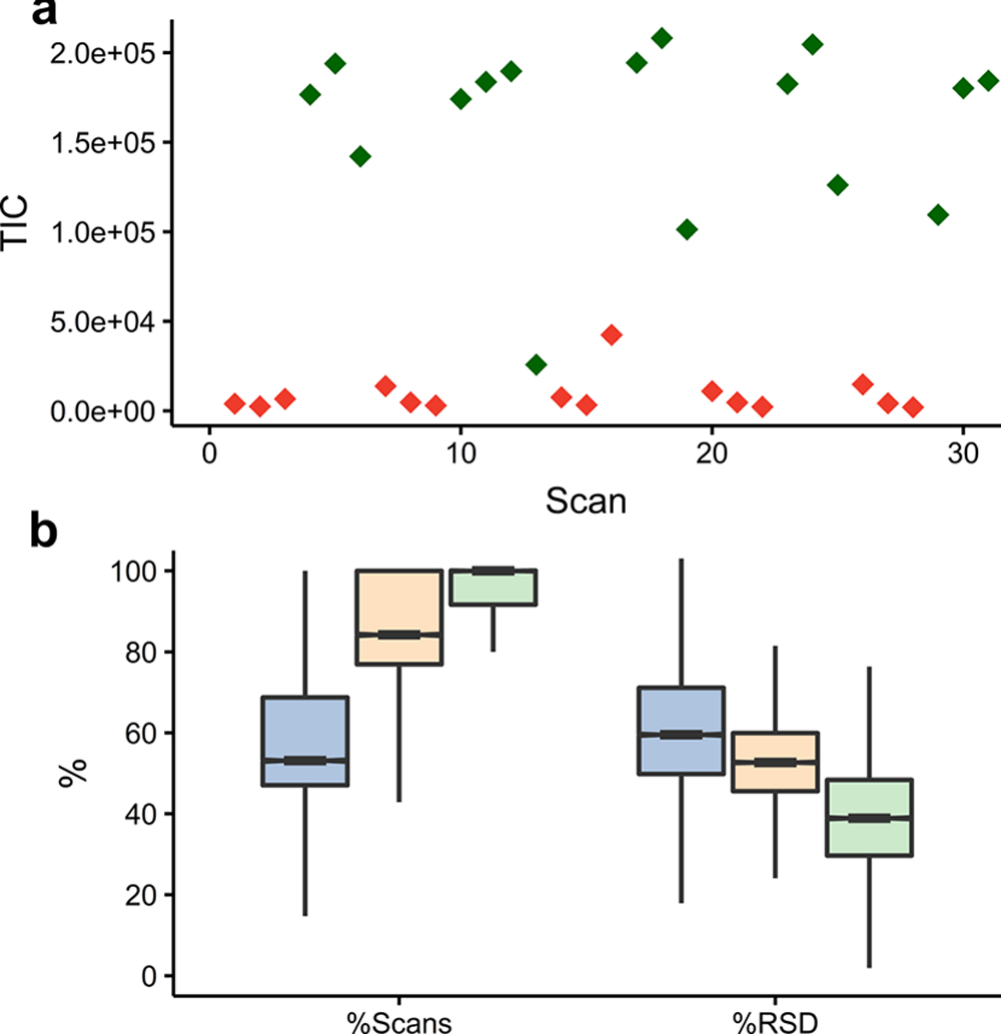
(a) Total ion current versus scan number showing “on-” (green) and “off-” (red) scans for AMI–MS metabolomics data acquisition of a representative HepG2 extract; (b) distribution of the percentage detectability of standards in scans across each sample, and the RSD of the intensities of the standards, comparing “all-scans” (blue), “on-scans” (yellow), and “on-scans-no-edge” (green) methods for extracting scan data from AMI–MS analysis of the standard sample set.

Raw data extraction sought to maximize the percentage of replicate scans that detected real signal, within each sample, and minimize the relative standard deviation (RSD) of the intensities of these features. Two factors were observed to influence the signal quality, first the switching of the charge cone polarity that generated on- and off-scan data and second our observation of “edge effects”, that is, reduced signal intensity in the first and/or last on-scan due to the temporal proximity of the polarity switch (Figure 2a). Consequently, three methods to extract the raw data were compared: (1) “all-scans”—the mean intensity of each feature was calculated across all on- and off-scans from the sample; (2) “on-scans”—the mean intensity was calculated from all on-scans only; and (3) “on-scans-no-edge”—the mean intensity was calculated from all on-scans that were not immediately preceded by or followed by an off-scan (illustrated in Figure S1). For the unusual case of only two consecutive on-scans (e.g., due to a short user-defined duty cycle), the single scan with the highest intensity was extracted. To undertake this comparison of extraction methods, first, an automatable decision threshold to assign a scan as “on” or “off” was required (Figure 2a), which was achieved through extensive manual interrogation of the raw data derived from the standard data set. Metabolite standard data were used to ensure that this fundamental processing step was optimized reliably, with the decision threshold set according to a hard signal-to-noise ratio (SNR) value that correctly extracted manually annotated *m/z* features of the standards, coupled with prior knowledge of the duty cycle timing. To account for a low number of high intensity features detected in the off-scan data, a second threshold was also optimized to allow an appropriate number of features with the SNR greater than the hard SNR threshold to be present in off-scans. Following optimization using the standard data set, the biological control data set was used to ensure robustness of the on-/off-scan labeling. Subsequently, the three methods to extract the raw data were compared.

### Optimized Filtering of *m/z* Features and Samples

Extensive *m/z* feature and sample-filtering capabilities originally developed to separate signal from noise in nESI–DIMS data^8^ were implemented and the relevant parameters were optimized for the novel AMI–MS metabolomics scan data. First, any features with an SNR (value optimized in this study, see below) less than a threshold value were discarded, termed “SNR filtering”; then, only those features present in at least a threshold percentage (value optimized in study) of the technical replicate analyses of each sample were retained, termed “replicate filtering”; and only those features in at least a threshold percentage (value optimized in study) of all samples in the study were kept, termed “sample filtering”. Missing intensity values occurred in the resulting data matrix, as occurs routinely in metabolomics data sets,^20^ so the percentage of missing values per sample was calculated to determine a threshold value (optimized in study) above which the sample spectrum was regarded as low quality and discarded. These parameter optimization procedures used the biological control data set to best represent a metabolomics study. The final feature filtering step calculated the RSD of the intensity of each remaining feature across all samples and discarded those that exceeded a threshold (value optimized in study). This “RSD filter” was based on the technical replicate data set.

### Normalization and Batch Correction

Intensity correction algorithms were applied to the AMI–MS data to enable reliable comparisons of samples given both the inherent nature of systematic intra- and interplate variation in mass spectrometry studies^21^ and the unusually large number of samples and 384-well plates comprising an AMI–MS study. First, the intra- and interplate intensity variations were characterized based on the measurement of the standards across the technical replicate data set and through principal component analysis (PCA) of distinct metabolic phenotypes in the toxicity data set. Then, a suitable two-step algorithm was implemented for effective normalization and batch correction of the data. In step one, probabilistic quotient normalization (PQN)^22^ was applied to each plate individually, using a plate-specific reference spectrum (calculated as the mean spectrum of a given plate) to ensure it was representative of the sample space. Next, the intensity of each *m*/*z* feature was batch-corrected by dividing the intensity values by a plate-specific coefficient for the given feature (calculated as the median intensity of the feature, for a given plate, divided by the grand median—equations given in Supporting Information).

### Cell Phenotype Filtering

Samples exhibiting high cell death in the HepG2 toxicity study (and therefore metabolic phenotypes that convey little about a drug’s MoA) were filtered from the metabolomics data matrix using a “cell phenotype filter”, which flagged (for removal) any exposure time/concentration drug treatment that induced a cell count below an optimized threshold value. The determination of those treatments that were cytotoxic was derived from the cell imaging data set collected from the lysis plates of the toxicity study, the optimized filter was then assessed by comparing the PCA of the filtered and unfiltered data from the toxicity study.

### Drug-Induced Metabolic Perturbations

The optimized processing steps, listed in Table S3 and discussed within the paper, were all applied to the toxicity data set using the associated cell imaging data, AMIMSpy and R v4.0. The only exception was that the RSD filter could not be applied to the toxicity data set as intrastudy quality control (QC) samples were not available due to constraints imposed by the automated extraction of 384-well plates. The MoA’s of the drugs in the toxicity study were investigated using PCA and Uniform Manifold Approximation and Projection (UMAP) analysis, and the concentration response of significant metabolites, determined from PCA loadings and analysis of variance (ANOVA), was visualized. Processing the toxicity data set was completed in <3 h utilizing 4 CPU cores in parallel and 16 GB RAM (Intel i7 CPU 3.40 GHz).

## Results and Discussion

### Extracting Raw Scan Data

Following manual interrogation of the standard data set, an SNR threshold of 15 was selected to distinguish on- from off-scans, since the majority of features in the off-scans had SNR < 15 and the majority of features in the on-scans were above this threshold.

Some features in the off-scans, however, had higher SNR (potentially caused by contamination from the ion transfer line), so a threshold number of such features (SNR > 15) that could be present within off-scans also had to be determined for more robust labeling of the scan types. A receiver operating characteristic (ROC) curve (Figure S3) identified that allowing three of these high intensity features within off-scans was optimal, giving a sensitivity of 0.91 (detecting on-scans as “on”) and specificity of 0.98 (off-scans as “off”).

Next, the three methods to extract the raw scan data were compared, with the evaluation based on which the method detected the metabolite standards in the most replicate scans and which yielded the lowest RSD values of the intensities of these standards (Figure 2b, using standard data set). A clear relationship was discovered, with low percentage occurrence correlating with the highest RSD values. The best-performing method was “on-scans-no-edge”, with a median occurrence of 100% and a median RSD of 38.90%, likely because it most effectively removed scans with any significant “off” character. These findings also provide an indication of the analytical reproducibility of the AMI–MS, with the median RSD ca. 2–3 times higher than can be achieved using nESI–DIMS,^8,21^ which is arguably an acceptable compromise for the >10-fold increase in sample throughput. The extraction of raw scan data in the remainder of this study used an SNR threshold of 15 (allowing up to 3 high intensity features in off-scans) and the “on-scans-no-edge” method.

### Filtering *m/z* Features and Samples

**Figure 3:**
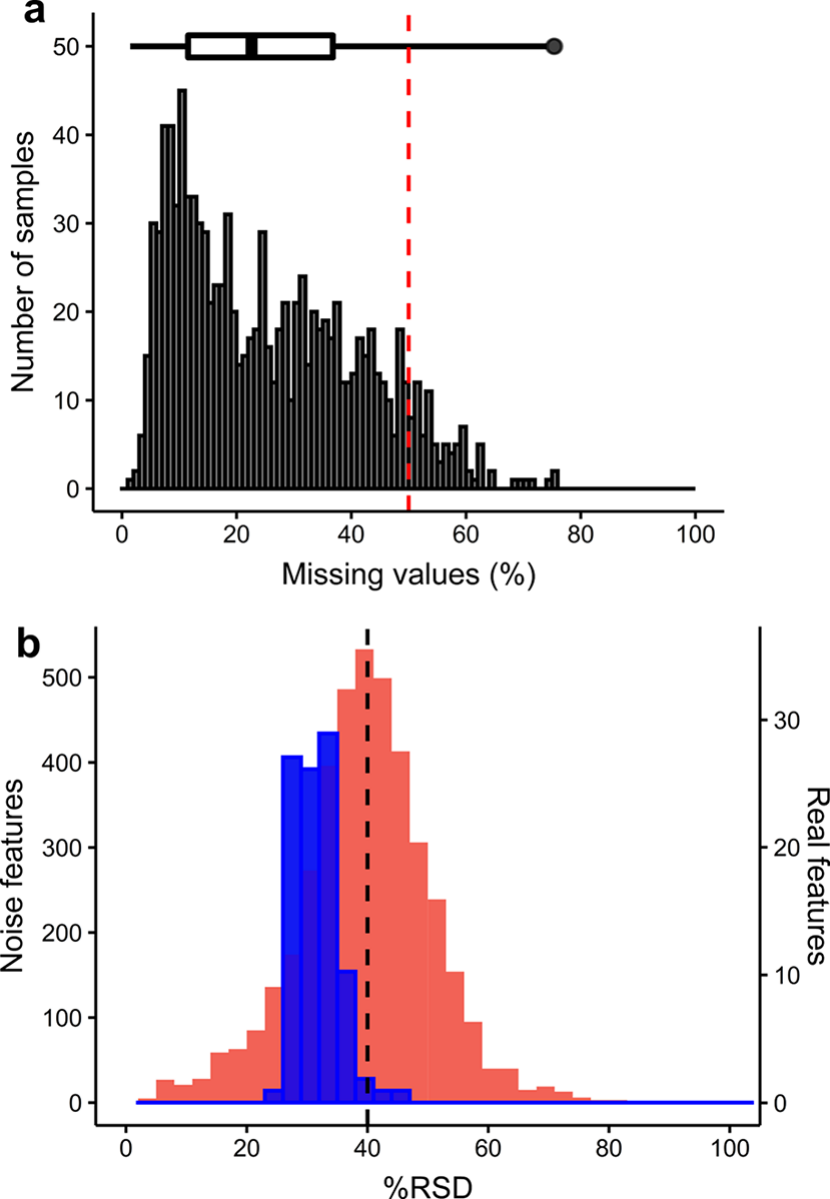
(a) Distribution of missing values in all samples from AMI–MS analysis of the biological control sample data set; (b) overlaid distributions of noise (red) and real features (blue) from AMI–MS analysis of the technical replicate data set. Note that y-axes are on different scales, and in both plots, the vertical lines indicate our optimized parameter for noise filtering.

The effects of multiple parameters associated with *m/z* feature and sample filtering were investigated to develop a robust signal-filtering workflow for the AMI–MS scan data. First, the effect of the “SNR filter” on feature count was investigated (Figure S4), providing no justification to deviate from the well-accepted norm of SNR > 3 to retain a signal.^8,23^ The optimal threshold for the “replicate filter” was 50% (Figure S7), which on average required features to be detected in a minimum of 3 out of 6 replicate scans per sample, based upon an analysis of the mean feature count per sample (i.e., sum of feature count across all samples divided by the number of samples). This value is lower than that used in nESI–DIMS, suggesting a less stable production of ions in AMI–MS related to nanoelectrospray ionization.^8^ Next, the effect of the “sample filter” on feature count was investigated (Figure S8), showing the expected decrease in feature count as the percentage threshold increased, and a value of 50% was selected based on a balance between not reducing the feature count too severely while attempting to retain only reliable, repeatedly observed features. Overall, using this combination of SNR, replicate, and sample-filtering thresholds, the feature count for the AMI–MS biological control data set was an order of magnitude lower than that routinely achieved using nESI–DIMS. However, a major cause of this apparently lower sensitivity of AMI–MS is the very low biomass of HepG2 cells being investigated, estimated at 3500 cells per well of a 384-well plate. In contrast, nESI–DIMS metabolomics studies have more typically analyzed ca. 100-fold greater biomass, helping to explain the apparent differences in sensitivity.^8^ The critical question is whether the AMI–MS-detectable metabolome, using a 384-well plate format, yields sufficient toxicological insights to achieve its screening mission, discussed below. Next, to filter out low-quality samples, the number of missing values per sample was assessed. A threshold of 50% was selected as this retained the majority (92%) of the biological control samples (Figure 3a). This threshold is lower than the >80% value often used within metabolomics experiments,^24^ owing to a lower sample filter threshold causing a greater number of missing values in the data.^8^ Optimizing the final filtering step revealed that an RSD threshold of 40% across the remaining high-quality samples was appropriate for the “RSD filter”, since it retained >95% of the real signal (SNR > 3, sample filter > 80%) while removing > 50% of the suspected noise features (SNR < 3, sample filter < 20%), shown by the distributions in Figure 3b. The noise features with unusually low RSD <40% (explained in Supporting Information) were removed by other components of the multistep filtering workflow that were purposely not applied for this RSD filter optimization.

### Normalization and Batch Correction

**Figure 4:**
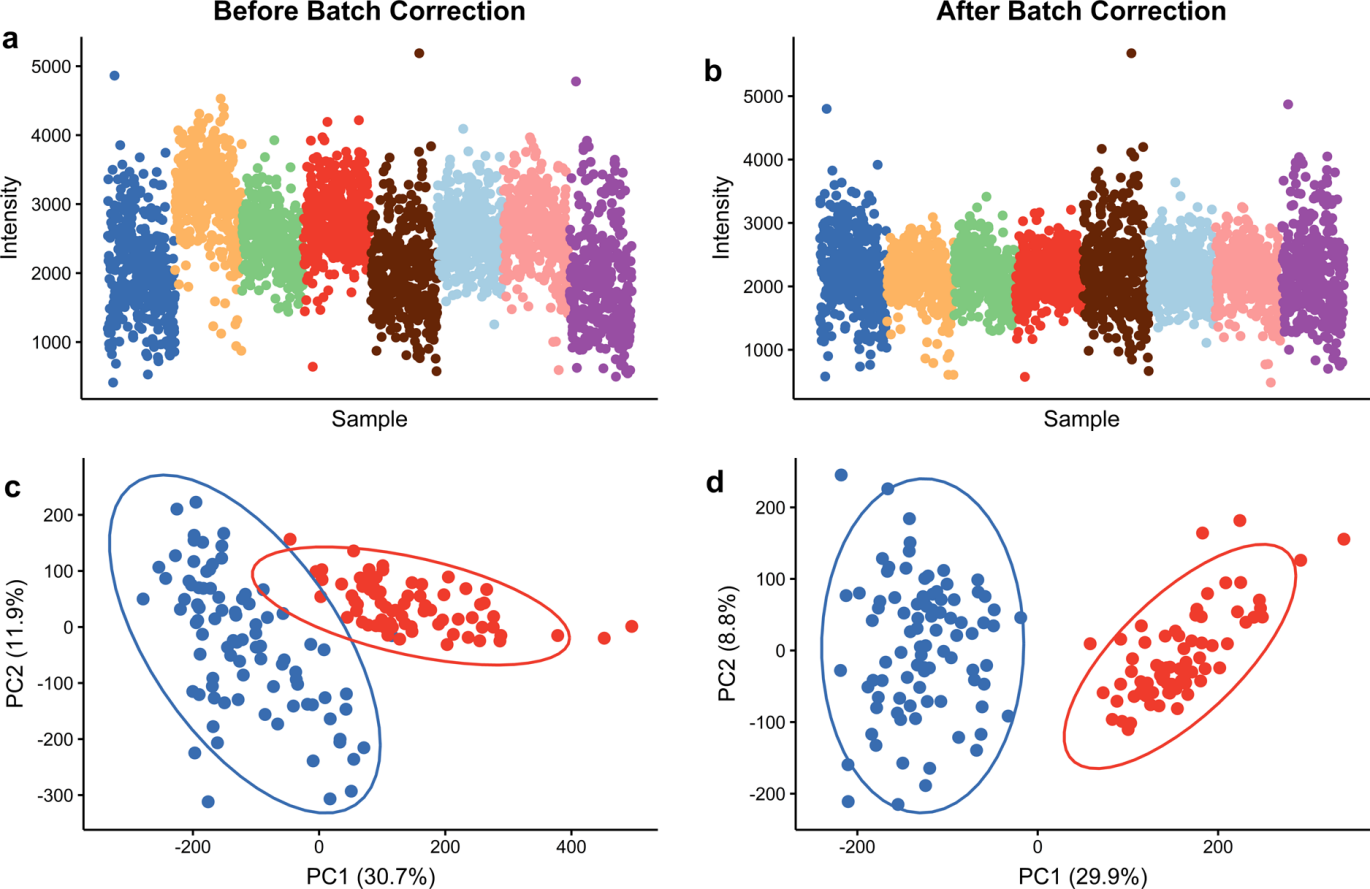
Scatter plot showing the intensity changes in the citrate internal standard from AMI–MS analysis across eight plates of the biological technical replicate data (a) before and (b) after batch correction, respectively, where each plate is colored differently. PCA score plot from AMI–MS metabolomics analysis of the DMSO-control (red) and death-control (blue) samples in the HepG2 toxicity study (c) before and (d) after batch correction. The batch correction algorithm reduces unwanted intensity variations and improves the separation between positive and negative control samples.

To minimize the intensity variance of features across replicate samples, PQN was applied, which reduced the median RSD from 29.4 to 24.8% in the technical replicate data set and is in line with metabolomics best practices.^10,25^ Even after this normalization, the intraplate variance is greater than that with traditional approaches, for example, Kirwan et al. reported a median RSD of <11.5% using nESI–MS metabolomics.^21^ However, AMI–MS metabolomics is proposed as a tool for UHT screening and it is not expected to match the analytical reproducibility of lower-throughput approaches. To characterize the interplate variance, the intensities of the internal standards in the technical replicate data set were tracked across eight plates (Figure 4a and Supporting Information); for example, the average intensity of citrate IS varied significantly across the plates, indicating that interplate variability needed to be addressed. The need to apply a batch correction algorithm was reinforced by PCA of the technical replicate data set (Figure S10), in which samples predominantly clustered based on the study plate. After applying the batch correction algorithm, the intensity variation decreased (Figure 4b and Supporting Information) and the PCA analysis was no longer dominated by interplate variance along the first principal component (Figure S10). The batch correction algorithm was also applied to a subset of the HepG2 toxicity study data set containing just the DMSO-control and death-control samples, two groups of samples that would be predicted to have distinct metabolic phenotypes, followed by PCA analysis. Figure 4c–d shows how the separation of these two groups of samples improves after batch correction, suggesting that interplate variance was present in the raw data. This batch correction algorithm is an important processing step when analyzing multiple plates, especially considering that AMI–MS metabolomics is capable of generating data from ca. 75 384-well plates in a single day.

### Cell Phenotype Filtering

**Figure 5:**
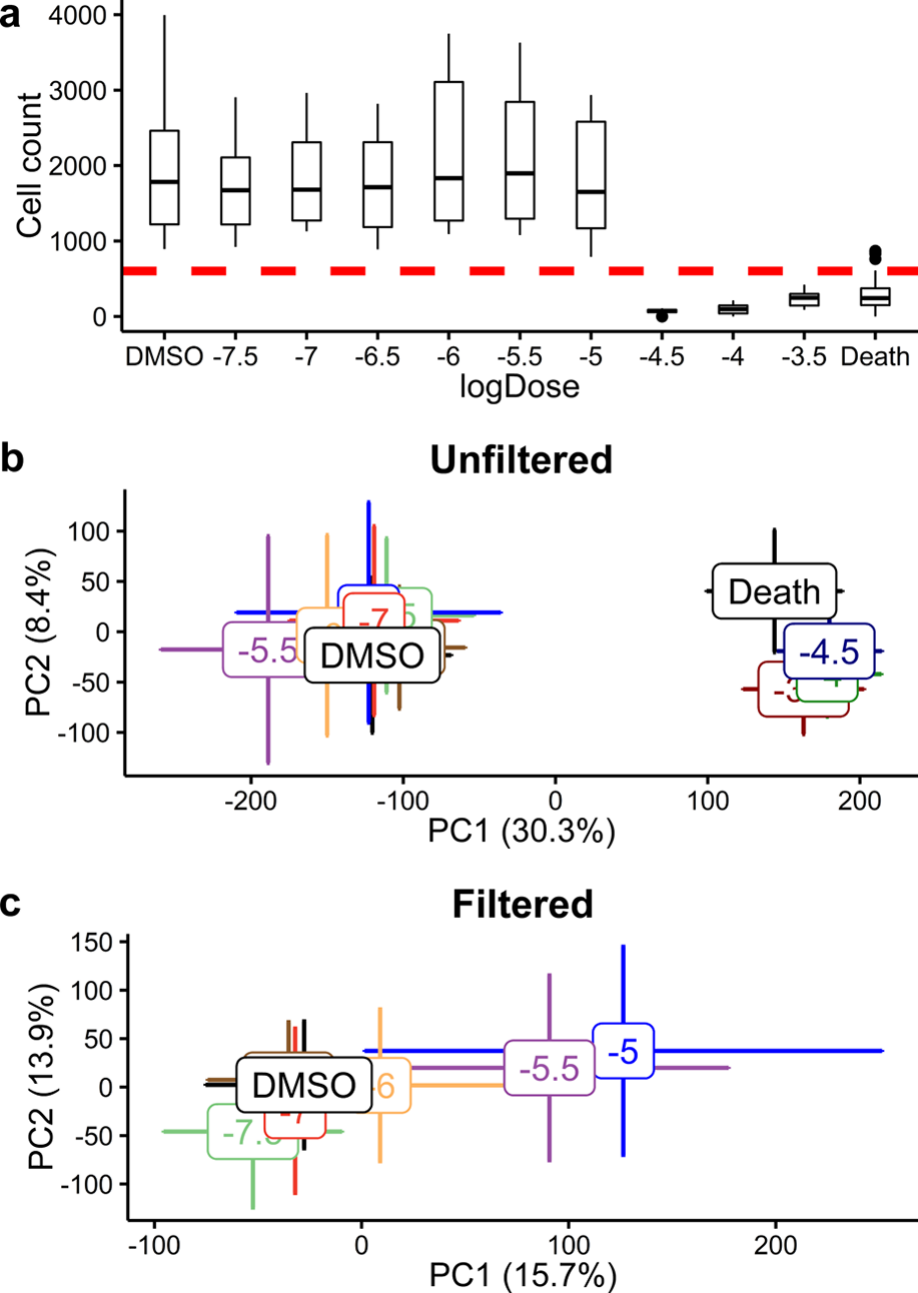
(a) Box plot representation of the cell count at each tamoxifen concentration from the cell imaging data, the red line indicates the cell phenotype filter threshold applied. PCA score plots from AMI–MS metabolomics analysis of the HepG2 toxicity study highlighting the metabolic responses to tamoxifen (b) before and (c) after cell phenotype filtering, respectively. The cell phenotype filter enables the more subtle concentration response associated with low tamoxifen exposure levels to be visualized.

A traditional toxico-metabolomics study typically requires an initial dose range-finding experiment to determine a small number of exposure concentrations for a subsequent smaller-scale omics investigation. This is due to the relatively low throughput and high costs associated with traditional metabolomics approaches. In contrast, UHT AMI–MS allows metabolomics to be readily applied to a large number of samples across a wide concentration range without an initial range-finding experiment. However, this time- and cost-saving benefit brings a new challenge of how to process widely differing metabolic phenotypes, from baseline metabolism to high cytotoxicity. We sought to remove the cytotoxic samples using a cell phenotype filter with a threshold of 602 cells, which corresponded to the 95th quantile of counts in the death-control samples (Figure S11). This threshold also retained all the DMSO-control samples and >80% of the drug-treated HepG2 samples, only rejecting samples that exhibited high cytotoxicity (Figure 5a). Applying cell phenotype filtering to the whole HepG2 toxicity study data set revealed previously hidden trends in the metabolic responses to some of the drugs. For example, prior to cell phenotype filtering, tamoxifen-treated samples were separated into two distinct clusters along the first principal component, corresponding to noncytotoxic and cytotoxic effects, but after filtering displayed a concentration response that was previously masked (Figure 5b,c). The associated PCA loading data (Figure S12) support the hypothesis that the major source of variance switches from cytotoxicity to a perturbation relating to MoA. This loading data also provide insights into the MoA of tamoxifen, discussed below. In contrast, deferoxamine showed no cytotoxic effects and the concentration response was evident without the need to employ cell phenotype filtering to reject any high-cytotoxicity samples (Figure S13).

### Drug-Induced Perturbations Revealed by AMI–MS Metabolomics

**Figure 6:**
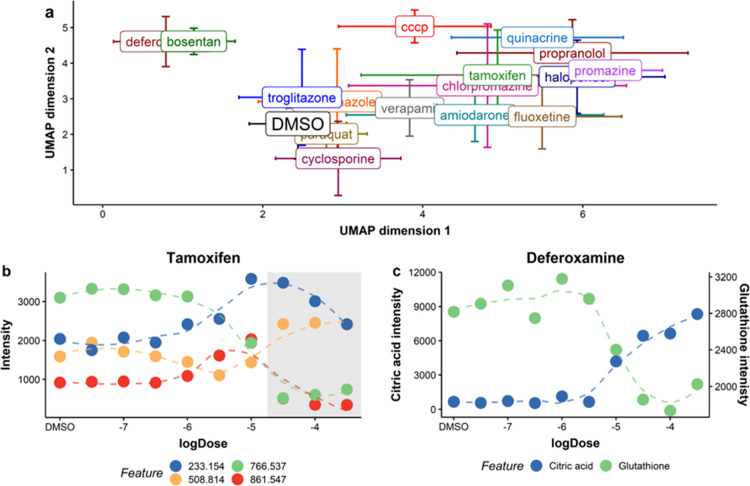
AMI–MS metabolomics analysis of the HepG2 toxicity study samples: (a) UMAP analysis of the highest noncytotoxic concentration of each of the 16 drugs, showing a clustering based on metabolic perturbations. Concentration–response relationships of key metabolite features (*m*/*z*) following exposure to (b) tamoxifen and (c) deferoxamine, respectively. Dashed lines are derived from smoothing functions, not dose–response models. Gray region in plot (b) indicates cytotoxic concentrations.

The AMI–MS metabolomics workflow was tested using the HepG2 toxicity study data set, comprising 2772 samples distributed across nine 384-well plates, representing the metabolic responses to 16 drugs. All steps of the workflow (Figure 1) were applied as optimized (Table S3), except for the RSD filter, due to the lack of intrastudy QC samples. Traditionally, these QC samples are generated by pooling an aliquot of every biological sample in a metabolomics study,^10^ which was not feasible here given the sole use of automated extraction methods, although in future studies, we recommend a strategy that is developed to generate and use intrastudy QC samples in AMI–MS. Following cell phenotype filtering, we first conducted an analysis using UMAP on only the highest noncytotoxic concentrations of each drug. This untargeted profiling revealed four distinct clusters of samples (Figure 6a; see also PCA score plot in Figure S14) pertaining to (i) DMSO-controls and drugs eliciting minimal metabolic responses, (ii) bosentan and deferoxamine, (iii) carbonyl cyanide 3-chlorophenyl hydrazon (CCCP), and (iv) a large cluster of drugs inducing a similar, significant metabolic effect on the right of the plot. The molecular perturbations driving each cluster were determined by conducting further analyses of each drug separately, focusing on metabolite features with high PCA loadings (Figures S12 and S13c) and based on univariate analysis (ANOVA) of individual metabolites across noncytotoxic concentrations (Figure S15). To illustrate the findings, Figure 6b shows the concentration responses of the most significantly perturbed features induced by tamoxifen, comprising changes in the lipidome. These perturbations are likely driven by phospholipidosis, a known MoA of the drugs within that UMAP cluster.^26^ The gray-shaded region in Figure 6b shows the cytotoxic sample space, where most of the metabolic effects are reversed, emphasizing the importance of studying responses over a wider concentration range and the value of cell phenotype filtering to focus only on noncytotoxic perturbations. As a second example, analysis of the effects of deferoxamine (Figure 6c) reveals a significant increase in citric acid abundance at higher concentrations coupled with depletion of glutathione, key indicators of tricarboxylic acid-cycle disruption and oxidative stress. These findings indicate how different perturbations to the detectable HepG2 metabolome, confirmed by dosing with proven liver injury drugs of known MoA’s, could be used to screen drug candidates for liver toxicity. One limitation of this UHT approach is that metabolite identification can only be achieved to metabolomics standard initiative level 3 given that only MS1 data are generated over such a short time scale.^27^ While this approach to data generation prevents robust biochemical inferences of toxicity mechanisms, this is not our proposed role for AMI–MS metabolomics. Instead, this UHT platform would be used to screen vast numbers of samples and utilize untargeted profiling to discover the relatedness of their metabolic phenotypes to drugs of known toxicity mechanisms. Traditional metabolomics approaches with higher metabolic specificity could be used to analyze selected cell extracts, depending on the study objectives.

## Conclusions

This study introduces signal-processing steps specific to AMI–MS data and adapts and optimizes traditional processing algorithms from an nESI–MS workflow, for this novel UHT metabolomics platform. We demonstrate that rejecting “off” and “edge” scans in AMI–MS data sets greatly improves data quality and that traditional noise-filtering approaches (including SNR cutoff, replicate filter, sample filter, missing value filter, and RSD filter) are compatible with this data type, following optimization. The implementation of normalization and batch correction algorithms had to be adapted, primarily due to the lack of intrastudy QC samples from the fully automated extraction methodology used in the AMI–MS experiment. Additionally, we implemented a data-dependent cell phenotype filter to aid the interpretation of noncytotoxic AMI–MS metabolic measurements. During optimizing the parameters in the AMI–MS workflow, we reported somewhat lower feature counts, increased technical variance, and lower confidence in metabolite annotations compared with the current state-of-the-art direct infusion mass spectrometry metabolomics approaches. However, despite these limitations of AMI–MS, we have demonstrated its unprecedented benefit of ultrahigh sample analysis, with HepG2 metabolomics data generated from a highly replicated exposure to 16 drugs across 9 concentrations—amounting to almost 3000 samples—in under 5 h. Analyses of these data using univariate and multivariate methods revealed metabolic changes consistent with the known drug-induced hepatotoxicity. This suggests that AMI–MS metabolomics could be deployed as an effective UHT metabolic screening tool in toxicology and other fields, analyzing ca. 2 orders of magnitude more samples within a given time period relative to the existing methods.

## Supporting Information Available

Experimental procedures; *m/z* alignment; normalization and batch correction; noise features with low RSD; list of standards; compounds and their metadata; processing parameters; AMI–MS schema; synthetic representation of extract scan algorithm; ROC curve; SNR filter plot; replicate scan filter plot; sample filter plot; PCA score plot of technical replicate data; boxplot representation of cell counts from cell imaging data; PCA loading plots from tamoxifen treatments; PCA and cell count from deferoxamine treatments; PCA score plot of all treatments; volcano plots for selected treatments; AMI–MS data sets and metadata described in this work, along with the processed and cell imaging data from the toxicity study are available at https://doi.org/10.5281/zenodo.4890974 (PDF).
