# Supplementary material for: Acoustic Mist Ionization Mass Spectrometry for Ultrahigh-Throughput Metabolomics Screening
Source: Anal Chem. 2021 Jun 22;93(26):9258–66. doi: 10.1021/acs.analchem.1c01616 (PMC8264826; doi:10.1021/acs.analchem.1c01616)
Supplement: Supplementary file 1 — ac1c01616_si_001.pdf [file ac1c01616_si_001.pdf]

Supporting Information for:

Acoustic mist ionisation - mass spectrometry (AMI-MS) for ultra-high throughput metabolomics screening

Matthew J. Smith,[a] Delyan P. Ivanov,[b] Ralf J. M. Weber,[a] Jonathan Wingfield[b] and Mark R. Viant*[a]

[a]School of Biosciences University of Birmingham, Edgbaston, Birmingham B15 2TT, UK;

[b]Mechanistic Biology & Profiling, Discovery Sciences, R&D, AstraZeneca, Cambridge, CB4 0WG, U.K

Table of contents:

Experimental Procedures 2
- Cell culturing 2
- Sample sets 2
- Data acquisition 2
- Data processing 3

m/z alignment: methods and optimization of parameters 3
Normalization and Batch Correction Algorithm equations 4
Investigation of noise features with low RSD 4
Supplementary tables 5
Supplementary figures 9
References 17

Experimental Procedures

Cell culturing

HepG2 C3A subclone cells were acquired before ATCC. They were STR-authenticated and mycoplasma tested for release for use in the assays. 500 mL Glucose-free Dulbecco's modified eagle medium (DMEM) with 4mM L-glutamine (Gibco, #11966-025), 50 mL foetal bovine serum (FBS, Gibco), 5 mL sodium pyruvate (100 mM, Sigma #S8636), 2.5 mL HEPES (1 M, Sigma #83264) and 10 mL galactose (0.5 M, Gibco #G5388) were used to prepare cell culture media.

Other reagents: Ammonium formate (Chromanorm,VWR, #84884.260) phosphate buffered saline (PBS, Sigma #D8662) and TrypLE Express (Gibco #12604-013) and Hoechst 33342 (Thermofisher Scientific #H3570) were used in subculturing and nuclear staining. HepG2 C3A cells were cultured on 175 cm^2 collagen-I-coated flasks (Corning BioCoat #356487) in routine cell culture and on 384-well poly-D-lysine coated plates (Corning BioCoat #356663) for imaging and metabolomics analysis.

Sample sets

Standards dataset. The standards dataset was prepared by aliquoting a 25:75 MeOH:H2O (v/v) solution of stable isotope-labelled and unlabelled standards from their stocks across a single 384-well plate. See Table S1 for a list of the consistently detected standards and their final concentrations.

Biological control dataset. The control dataset was formed of biological replicate samples from HepG2 lysates. HepG2 cells were seeded across a single 384-well plate at 3500 cells/well in galactose media (40µL) using a Multidrop Combi (ThermoFisher Scientific) with a standard cassette at low speed. Cells were then cultured for 48h at 37 °C, 5% CO_2 without any treatment. The cells were then washed 6 times with freshly prepared 150 mM ammonium formate buffer (pH=7.2-7.4) using a Biotek EL406 plate washer. The excess ammonium formate after the last wash was removed using a centrifugal washer (Bluewasher). The cells were then lysed and quenched in plates with chilled methanol (t = -80 to -40 °C, V=15 µL), heat-sealed and placed in a -80 °C freezer for at least an hour. Plates were taken out of the freezer and a solution of stable isotope-labelled standards was dispensed in each well and the plates incubated on a plate shaker for 20 mins (900 rpm). An aliquot of the lysate (25µL) was then transferred into an echo-compatible plate (Labcyte P200) prefilled with deionised water (25 µL), bringing the total volume to 50 µL and final ratio of MeOH:Water (25:75 v/v).

Technical replicates' dataset. The technical replicates dataset was derived from a single HepG2 lysate from cells cultured in the same conditions as the control dataset. Prepared in bulk (175 cm^2 collagen-I coated flasks) from untreated cells, lysed after washing with ammonium formate, with added stable isotope-labelled standards and MeOH:Water (25:75 v/v). Samples for this dataset were dispensed in eight 384-well plates across 3 days.

Toxicity study dataset. For the toxicity dataset cells were seeded as in the biological control dataset but were left to attach for 24h and then treated using an automation platform (Star6) consisting of a cell culture incubator (MolecularDevices, SteriStore), robotic arm, and the Echo 555 liquid handler (Labcyte) and placed back in the incubator for 24h. After incubation the washing and lysis steps follow the same protocol as for the biological control dataset.

The samples were treated with 16 compound stocks (50 mM in DMSO of each of compounds listed in Table S2) at nine half-log spaced concentrations with highest compound concentration of 316µM and final DMSO concentration 1% (v/v) as previously described.[1] Each compound concentration was tested in nine separate plates containing two technical replicates per plate across 3 days (n=18). Each destination cell plate included 10 technical replicates from both negative-control (1% DMSO) and positive-control (316µM chlorpromazine). Compound concentrations and controls were randomly distributed on the plate.

Data acquisition

AMI-MS data. A 3 kV voltage was supplied to the charging cone above the test well to induce a charge gradient within the sample. This resulted in an accumulation of negative charge on the surface of the liquid which generated a mist of nL sized droplets carrying the negative charge when the acoustic wave was pulsed through the sample at a frequency of 1400Hz. To discharge the well the polarity of the voltage was inverted for every 10 nL packets of

sample dispensed. From a total volume of 100 nL dispensed per sample 50 nL of sample carried negative and 50 nL carried positive ions

The droplet mist reached the Xevo G2-XS quadrupole time-of-flight mass spectrometer (Waters Wilmslow, UK) through a custom-built heated capillary (250 °c) into the source (100 °c). Desolvation of the droplet mist was achieved with the heated capillary, source and a cone gas flow of 50 L/h. The collision energy was set to 6eV and the detector was set to scan for 0.08 sec with an inter scan time of 0.014 s which resulted in an average of 3 scans per 10 uL package polarity switch. The mass range from 50 to 1200 Da was scanned with typical mass resolution of 25,000 full width of a peak at half maximum (FWHM). The ADC sample frequency was 6 GHz with a pusher frequency of 60 µsec and a pusher width of 1.5 µsec. The AMI-MS set-up is shown in Figure S1.

Cell imaging data. Since nuclear cellular DNA remained attached to the poly-D-lysine plates after lysis it was possible to perform a direct cell count in the lysis plates. Nuclei were stained with Hoechst 33342 in PBS (1:5000) dispensed with a Multidrop Combi at low speed. Plates were incubated for 20 mins at room temperature or stored at 4 °C for 24h. Nuclei were counted at 4x magnification using an automated fluorescence microscope CellInsight (ThermoFisher Scientific) and a dynamic threshold algorithm.

Data processing

For each processing step the relevant raw AMI-MS data was peak-picked using MassLynx v4.2 before being converted to .mzML format using MSConvert (freeware, http://proteowizard.sourceforge.net/). In parallel the metadata was parsed using an 'MS-Parser' tool provided by Waters to create .txt files which associated scans to the specific wells on the 384-well plates. These 2 files were taken as input by AMIMSpy (https://github.com/computational-metabolomics/amimspy) and the processed .txt data matrices output was further processed and analysed by a combination of in-house R scripts and StructToolbox[2] (https://github.com/computational-metabolomics/structToolbox).

m/z alignment: methods and optimization of parameters

Hierarchical clustering was used to group *m/z* features pertaining to the same metabolite in different scans and samples within the sample sets. The clusters were defined by cutting the dendrogram at a tolerance (ppm) which gave confidence the *m/z* features within the cluster were from the same metabolite. The appropriate tolerances for (i) replicate scans within a sample and (ii) samples within a sample set, related to the precision of the instrument at the time of acquisition so they were defined based on measuring the *m/z* errors from the stable isotope-labelled and unlabelled standards in the standards dataset. The standards data was used to ensure only reliable features were considered since metabolites in the biological sample were hard to distinguish given only MS1 data was available.[3]

To define the suitable precisions values in an objective and repeatable manner first the features from replicate scans within a sample were aligned (align scans) and then samples (align samples) as follows.

Align scans. For all replicate scans within each sample the *m/z* error of each standard (stable isotope-labelled and unlabelled standards from Table S1) was calculated - yielding an expected normal distribution (Figure S4a and S4b). The *m/z* precision at the time of analysing each sample was calculated as the standard deviation of the errors (ppm) from replicate scans within the sample multiplied by 2; which ensured 95% of standards were aligned to the correct *m/z* cluster, whilst maintaining purity of the feature. The distribution of the *m/z* precision values from each sample in the sample sets (Figure S4c) was used to find a global value. The global align scans tolerance value used to align scans within all the samples in the sample sets was defined as the value which captured 99% of the *m/z* precision values from the individual samples (99th quantile). For this work it was 20 ppm.

Align samples. After the alignment of scans within each sample; the *m/z* error of each standard in each sample across all the samples in the standards dataset was calculated - yielding an expected normal distribution (Figure S5). The *m/z* precision for aligning samples was derived from calculating the standard deviation of these errors multiplied by 2. The align samples tolerance was set to a slightly larger value to account for matrix effects in the biological samples.[4]

We propose a standard mixture akin to the system suitability QC sample[5] is acquired at the beginning and end of future AMI-MS experiments to evaluate the precision of the instrument during metabolomics studies in this way. This can also be used to assess other quality metrics, however that is beyond the scope of this work.

Normalization and Batch Correction Algorithm equations

The two-step algorithm for effective normalization and batch correction of the metabolomics data.

1. Probabilistic quotient normalisation[6] was applied to each plate individually, using a plate-specific reference spectrum (calculated as the mean spectrum of a given plate).
2. The intensity of each *m/z* feature was batch corrected by; (i) finding the plate-specific coefficient for the given feature (Equation 1) then (ii) dividing the uncorrected intensity values by the coefficient (Equation 2).

$$Coefficient_{plate,feature} = \frac{Batch\ median_{plate,feature}}{Grand\ median_{feature}} \qquad \text{Equation 1}$$

$$Corrected\ intensity_{sample,feature} = \frac{Initial\ intensity_{sample,feature}}{Coefficient_{plate,feature}} \qquad \text{Equation 2}$$

- $Batch\ median$ refers to the median intensity of a given feature across a given plate
- $Grand\ median$ refers to the median intensity of a given feature across all plates
- $Initial\ intensity$ refers to the uncorrected intensity of a feature in a given sample
- $Corrected\ intensity$ refers to corrected intensity of a feature in a given sample

Investigation of noise features with low RSD

In our work we found that some suspected noise features appeared to have a low RSD - a counter intuitive phenomenon that arose multiple times. The reason for this was briefly investigated and could be explained by the intensity distribution of these noise features with $\overline{SNR}$ ca. 3 (the SNR filter threshold used) being limited to only the samples or scans that showed a high SNR (SNR > 3) for the feature. In this scenario the RSD of a feature's intensity values is only calculated on the subsection of its entire intensity distribution - where the feature has SNR > 3 - which would synthetically decrease the RSD.

The SNR distributions of noise features and stable isotope-labelled standards across all the biological technical replicate samples (Figure S8a and S8b) supports this theory. The SNR values of most of the (low intensity) noise features across the sample set were < 3 and so were removed by the SNR filter. Whereas the standards had much greater SNR, so there were very few samples where the SNR of a features was < 3.

Additionally, the effect that increasing the SNR filter from 0 to 5 had on the intensity RSD of those noise features (and stable isotope-labelled standards) was investigated (Figure S8c and S8d). The RSD of the standards were unaffected given that very few feature intensity values had SNR < 5 so their intensity values were not removed, but the RSD of noise features decreased significantly with the applied SNR threshold; indicating that increasing SNR thresholds further limited the distribution of intensity values across the sample set for these noise features, thus reducing the RSD.

Supplementary Tables

Table S1. List of the standards that were spiked into the sample sets analysed as part of this work.

Standard Name	Standard type	Datasets	Final conc. µM
Aspartate_IS	Stable isotope-labelled calibrant	Standards dataset<br>Control dataset<br>Technical replicates dataset<br>Toxicity dataset	12.5
Citrate_IS	Stable isotope-labelled calibrant	Standards dataset<br>Control dataset<br>Technical replicates dataset<br>Toxicity dataset	0.5
Glutathione_IS	Stable isotope-labelled calibrant	Standards dataset<br>Control dataset<br>Technical replicates dataset<br>Toxicity dataset	1.7
Cholic acid_IS	Stable isotope-labelled calibrant	Standards dataset<br>Control dataset<br>Technical replicates dataset<br>Toxicity dataset	1.0
ATP_IS	Stable isotope-labelled calibrant	Standards dataset<br>Control dataset<br>Technical replicates dataset<br>Toxicity dataset	0.8
15:0-18:1(d7) PG_IS	Stable isotope-labelled calibrant	Standards dataset<br>Control dataset<br>Technical replicates dataset<br>Toxicity dataset	0.02
Aspartic acid	Unlabelled standard	Standards dataset	100
Citric acid	Unlabelled standard	Standards dataset	2
Glutathione	Unlabelled standard	Standards dataset	6.67
Deoxycholic acid	Unlabelled standard	Standards dataset	1
ATP	Unlabelled standard	Standards dataset	3.33
ADP	Hydrolysis product of unlabelled standard	Standards dataset	

AMP	Hydrolysis product of unlabelled standard	Standards dataset	
UDP-N-Acetylhexosamine	Unlabelled standard	Standards dataset	1
Oxidised glutathione	Unlabelled standard	Standards dataset	1
L-Carnitine_IS	Stable isotope-labelled standard	Standards dataset<br>Control dataset<br>Technical replicates dataset	1.1
Octanoyl_L-carnitine_IS	Stable isotope-labelled standard	Standards dataset<br>Control dataset<br>Technical replicates dataset	0.3
ADP_IS	Hydrolysis product of stable isotope-labelled standard	Standards dataset<br>Control dataset<br>Technical replicates dataset	
AMP_IS	Hydrolysis product of stable isotope-labelled standard	Standards dataset<br>Control dataset<br>Technical replicates dataset	

Table S2. Compounds used in the HepG2 toxicity dataset and metadata about their; intended use, drug induced liver injury (DILI) potential, mechanism, lesions, phospholipidosis potential (PLD) and mitochondrial impairment.

**Compound**	**Intended use**	**DILI severity category**	**DILI category**	**Mechanism**	**Pattern (Type of lesion)**	**PLD**	**Mitochondrial impairment**
quinacrine	antimalarial	3	Pos	NA	hepatocellular	TRUE	NA
ketoconazole	antifungal	1	Pos	NA	mixed	TRUE	1
haloperidol	antipsychotic	3	Pos	NA	mixed	TRUE	1
deferoxamine	chelating agent for iron toxicity	4	Neg	Redox	NA	FALSE	NA
paraquat	herbicide	NA	NA	Redox	NA	TRUE	NA
fluoxetine	antidepressant (SSRI)	3	Pos	NA	hepatocellular	TRUE	1
chlorpromazine	antipsychotic	3	Pos	NA	cholestatic	TRUE	1
cyclosporine	immunosuppressant	3	Pos	NA	cholestatic	NA	NA
propranolol	beta blocker	3	Pos	NA	hepatocellular	TRUE	NA
promazine	antipsychotic	3	Pos	NA	cholestatic	TRUE	NA
amiodarone	antiarrhythmic	1	Pos	PLD	mixed	TRUE	1
verapamil	calcium channel blocker	3	Pos	NA	mixed	TRUE	NA
cccp	oxidative phosphorylation uncoupler	NA	NA	Uncoupler	NA	NA	1
troglitazone	antidiabetic	1	Pos	NA	mixed	NA	1
bosentan	dual endothelin receptor antagonist	1	Pos	NA	cholestatic	NA	1/0
tamoxifen	estrogen receptor modulator	2	Pos	NA	mixed	TRUE	1

Table S3. List of processing steps and optimised parameters used for processing the HepG2 toxicity dataset.

**Pre-processing step**	**Parameter for toxicity dataset**
Labelling on scans	≥ 3 peaks with SNR > 15
Method for extracting relevant scan data	on scans no edge
SNR threshold	3
m/z precision (scans)	20 ppm
m/z precision (samples)	20 ppm
Replicate scan filter	50%
Sample filter	50%
Missing value filter	50%
RSD filter (intra-study QCs)	Not used due to limitations associated with generating representative QC samples
Interplate variability	Implement platewise batch correction algorithm
Cellular phenotype filtering	Retain samples with cell count > 602

Supplementary Figures

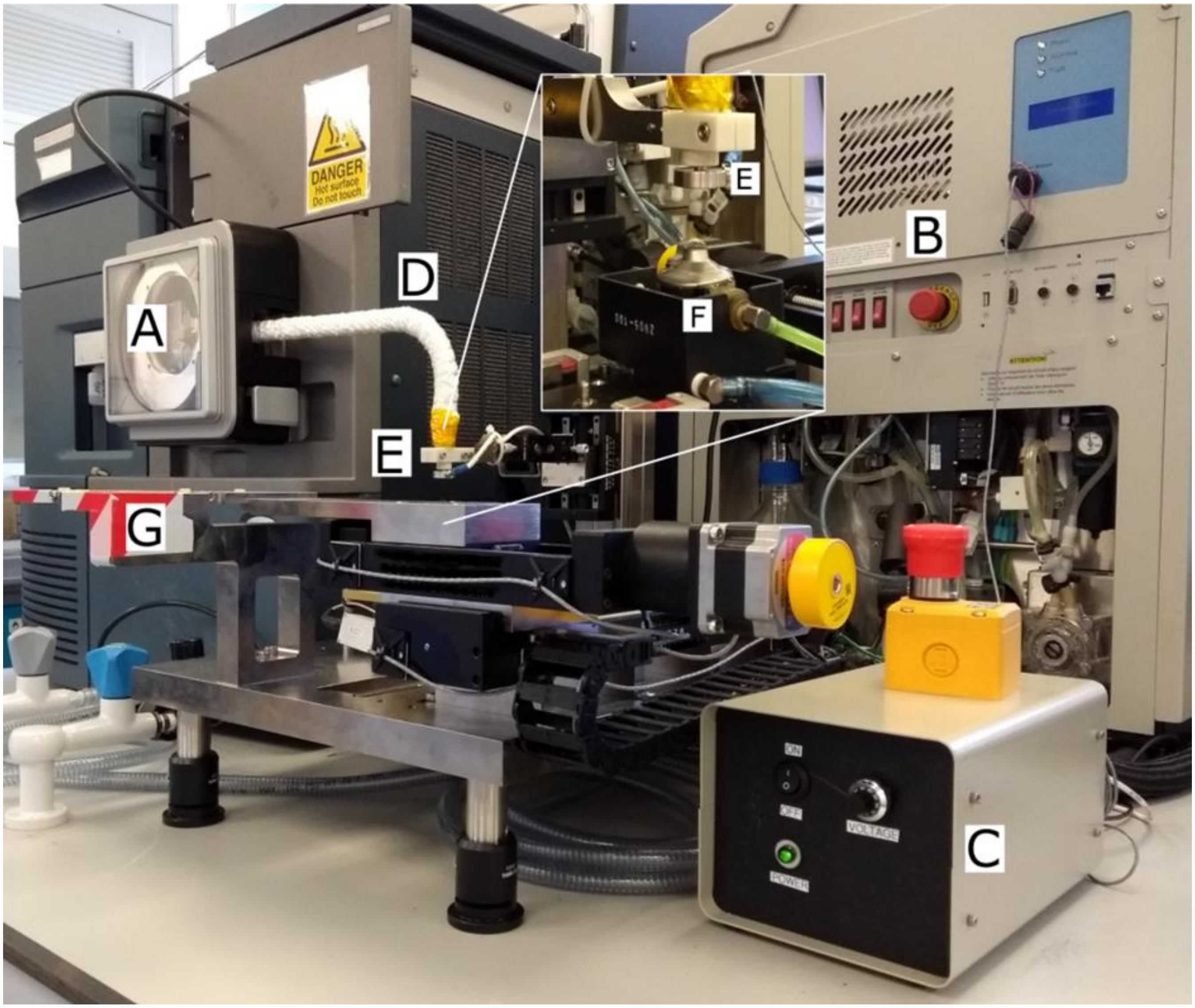

Figure S1. Picture showing the AMI-MS set-up (a) Waters Xevo G2-XS source; (b) Echo 555 electronics control unit; (c) Hv power supply; (d) heated capillary "transfer optic"; (e) Charging cone connected to Hv supply; (f) acoustic transducer assembly (from inside the Echo 555 unit).

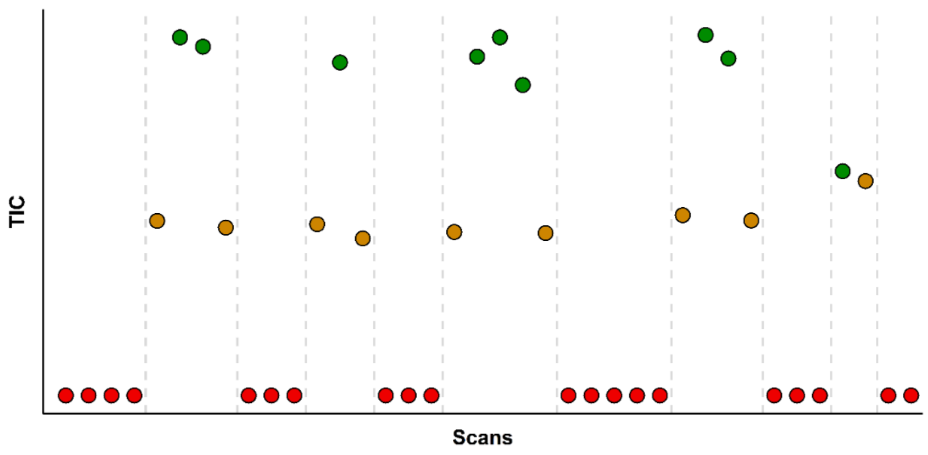

Figure S2. Synthetic data to show how our proposed algorithm for selecting high quality scan data ('on scans no edge') deals with different AMI-MS duty cycles. The points refer to off-scans (red), on-scans with edge effects (orange) and on scans without edge effects (green) – only the green scans are taken forward.

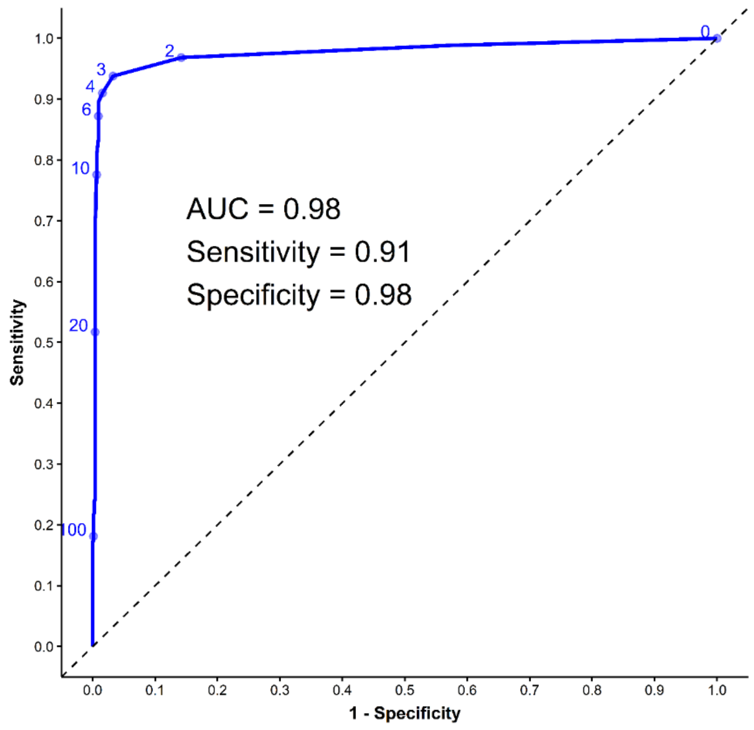

Figure S3. Receiver operating characteristic (ROC) curve showing the sensitivity and specificity of identifying on scans from 100 randomly selected samples from the AMI-MS biological controls dataset with manually curated ground truths (based on TIC v scan number plots and prior knowledge about the duty cycle). The curve is based on a hard SNR threshold of 15 and cuts relate to the threshold number of features that must have SNR > 15 to be labelled as an 'on scan'. The values for sensitivity and specificity relate to the optimal number of features (3) with SNR > 15 this model predicts.

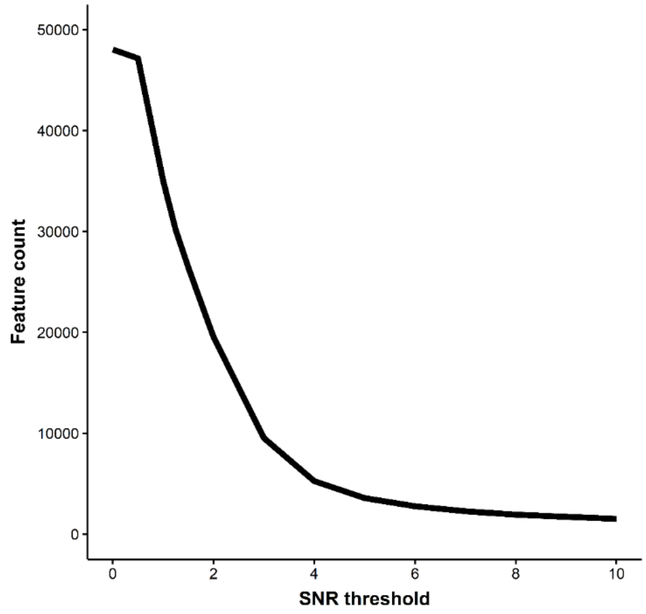

Figure S4. Effect of increasing SNR filter threshold on feature count in the AMI-MS biological control sample set.

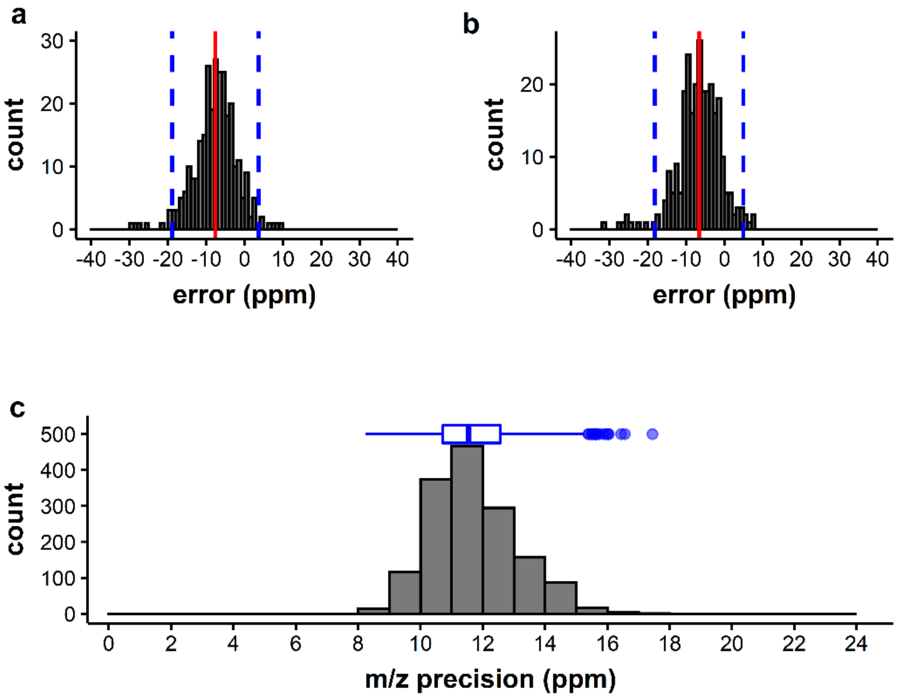

Figure S5. Mass error distribution from stable isotope-labelled and unlabeled standards measured from replicate scans of randomly selected samples (a) and (b) within the standards dataset following AMI-MS analysis. The red and blue vertical line represents mean error and ±2 standard deviations (m/z precisions) of the errors respectively. (c) Distribution of the individual *m/z* precision values for each sample across all the samples in the standards dataset.

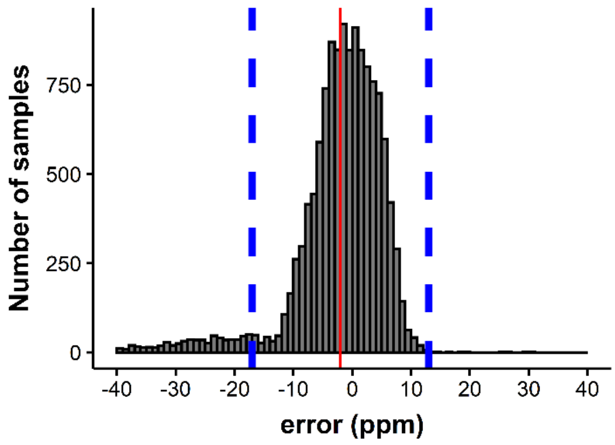

Figure S6. Distribution of stable isotope-labelled and unlabeled standards mass errors from all samples in the standards dataset. The red and blue vertical line represents mean error and ±2 standard deviations (m/z precisions) of the errors respectively.

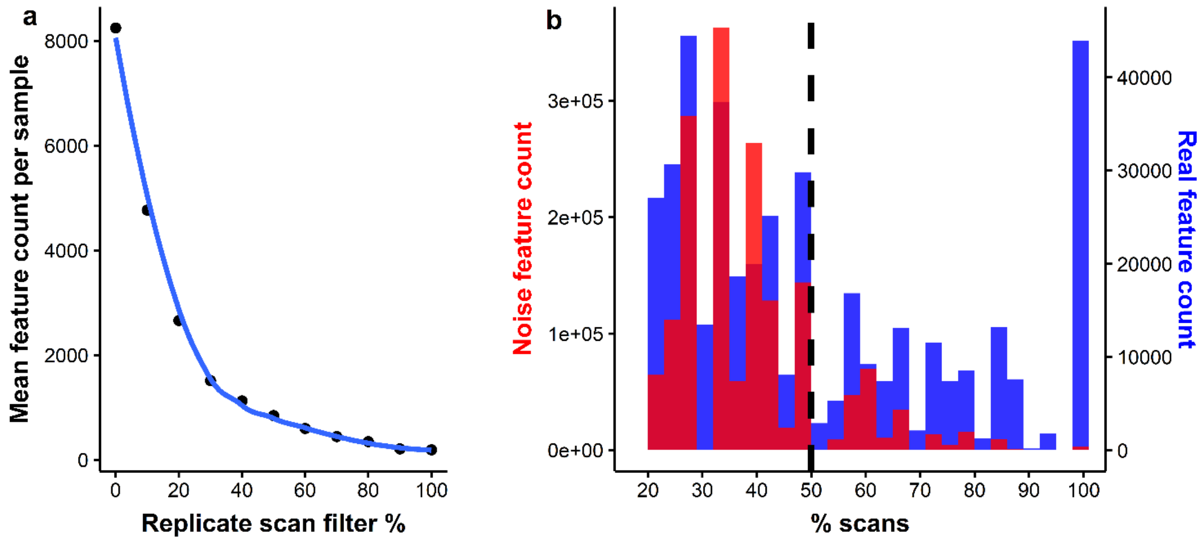

Figure S7. (a) Effect of increasing replicate scan filter on the mean feature count per sample in the AMI-MS biological control sample set. (b) Distribution of the percentage of replicate scans features are detected in for real metabolite features (blue) and noise (red) across all the biological control samples. Real features were determined as features with SNR > 10 and noise features with SNR < 3. It is likely that readily ionisable noise features are labelled as real features given the simplicity of the labelling approach, however the observed trend was suitable for determining a reliable threshold in this work. The vertical black line at 50% shows the optimised value given most 'noise features' are in fewer scans and a significant number of 'real features' are retained.

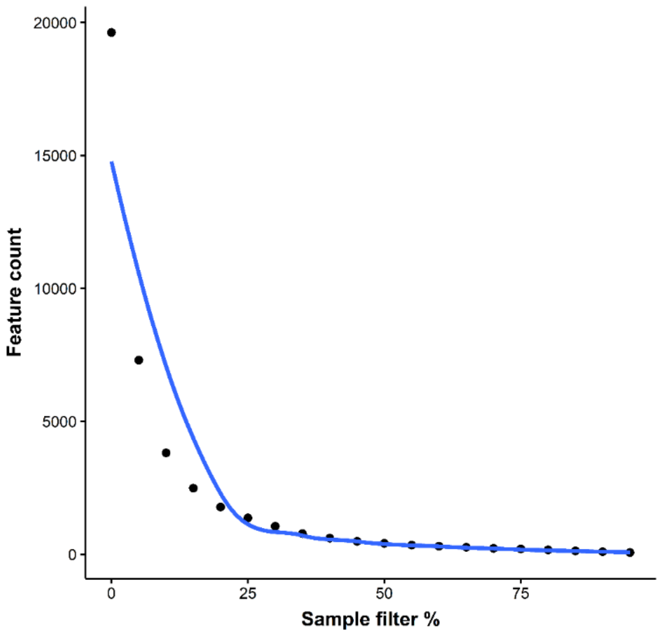

Figure S8. Effect of increasing sample filter on feature count in the AMI-MS biological control sample set.

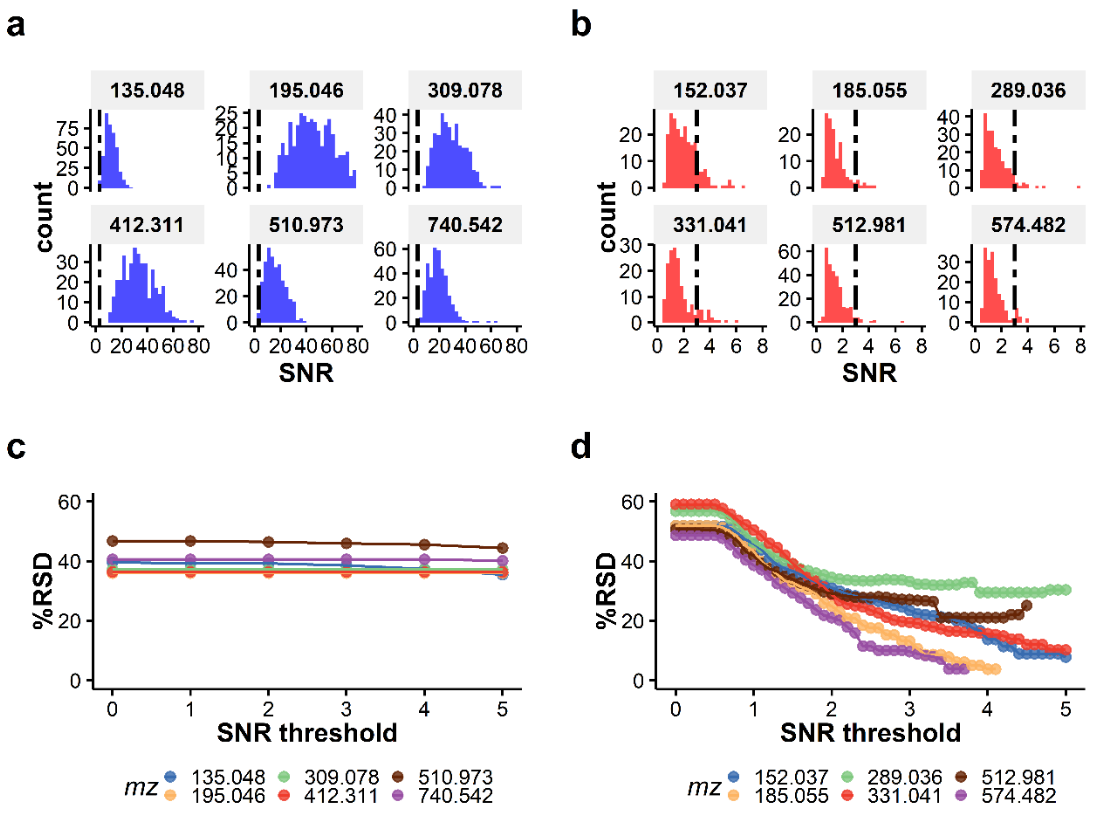

Figure S9. (a) and (b) Distribution of SNR of stable isotope labeled standards and noise features respectively from AMI-MS analysis of samples in the biological technical replicate sample set. The black vertical lines represent the SNR filter and highlights that noise features are removed from many samples due to having SNR <3, leaving a limited distribution of intensity values for these features across the sample set. (c) and (d) plot the change in RSD as a function of applied SNR threshold from the same analyses for stable isotope labeled standards and noise features respectively.

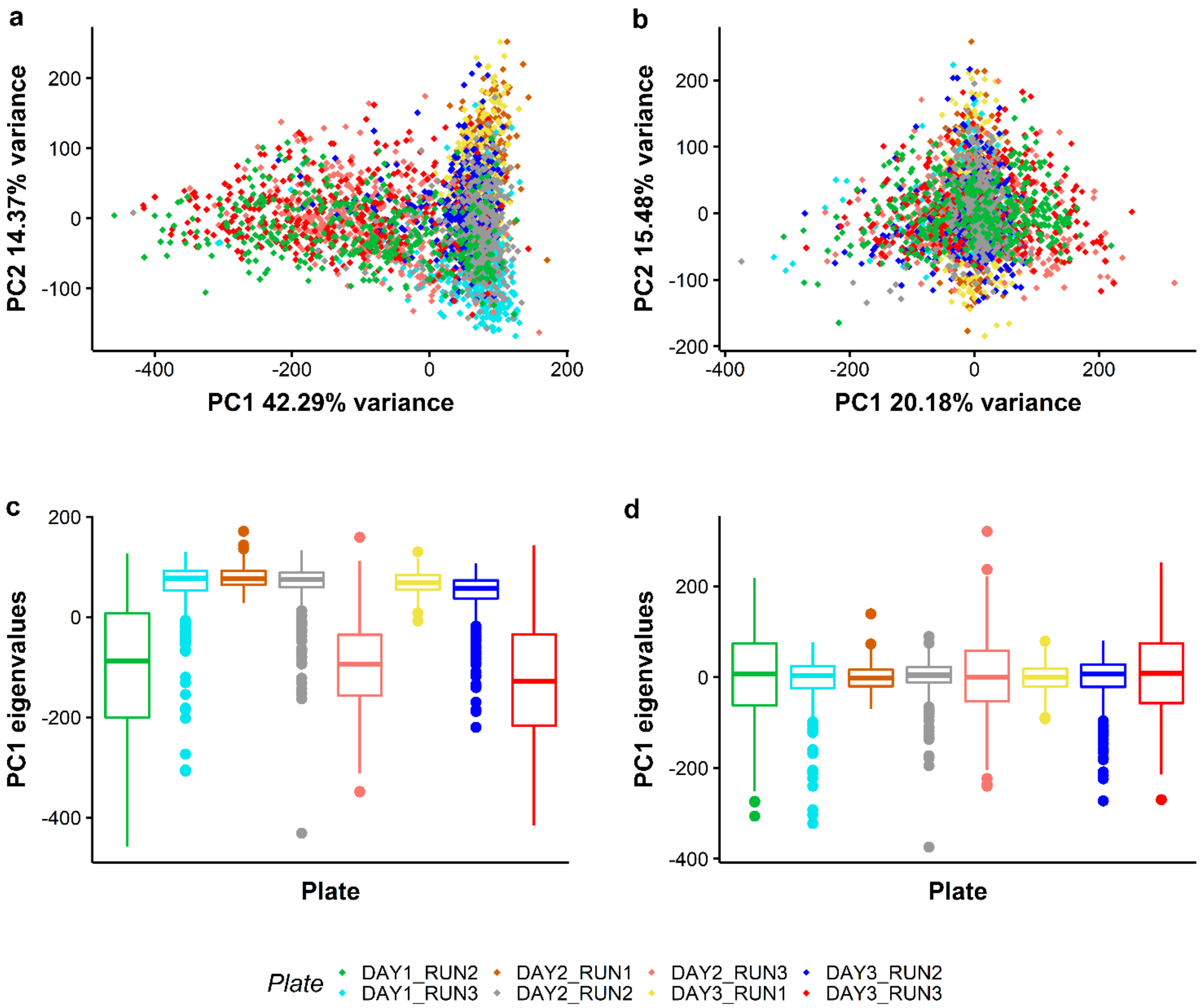

Figure S10. PCA scores plots from AMI-MS analysis of the biological technical replicates data before (a) and (c) and after (b) and (d) batch correction. An alternative boxplot representation of principal component analysis (PCA) scores is shown (c) and (d) for to aid visualisation of the reduced interplate variance observed along PC1 following batch correction.

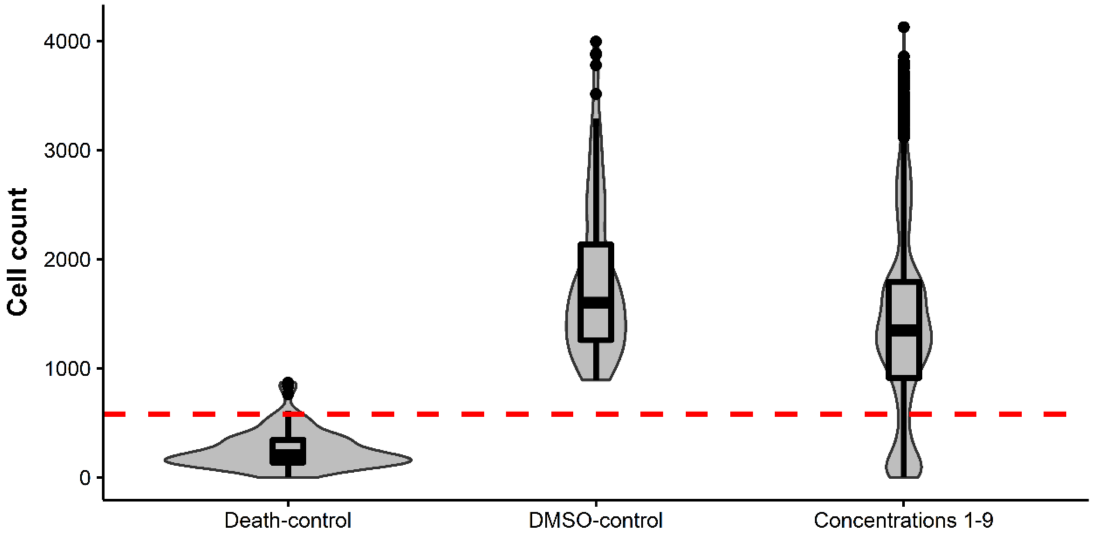

Figure S11. Distribution of cell count across; death-control, DMSO-control and all treatments (half log concentrations 1-9) samples from the cell imaging data. The red line gives the optimised cell phenotype filter; defined as the 95th quantile of cells in the death-control samples (602 cells).

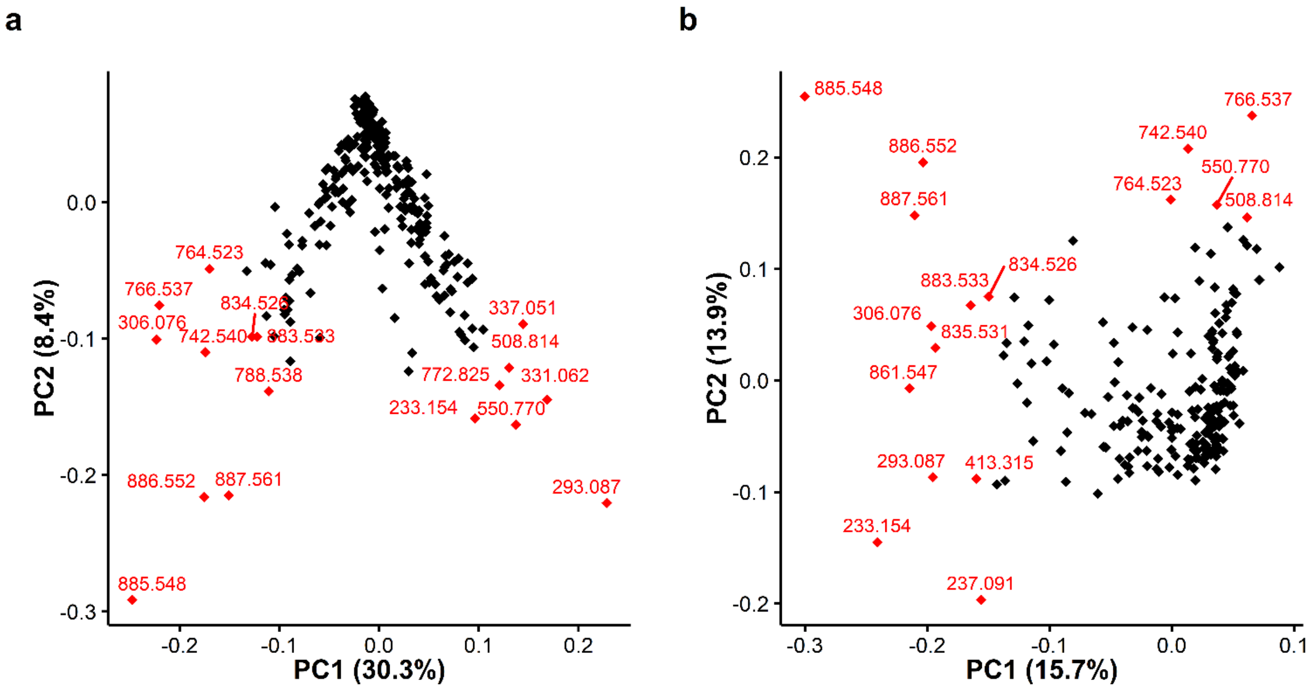

Figure S12. PCA loadings plots from AMI-MS metabolomics analysis of the HepG2 toxicity study highlighting the metabolic responses of tamoxifen treatment before (a) and after (b) cell phenotype filtering.

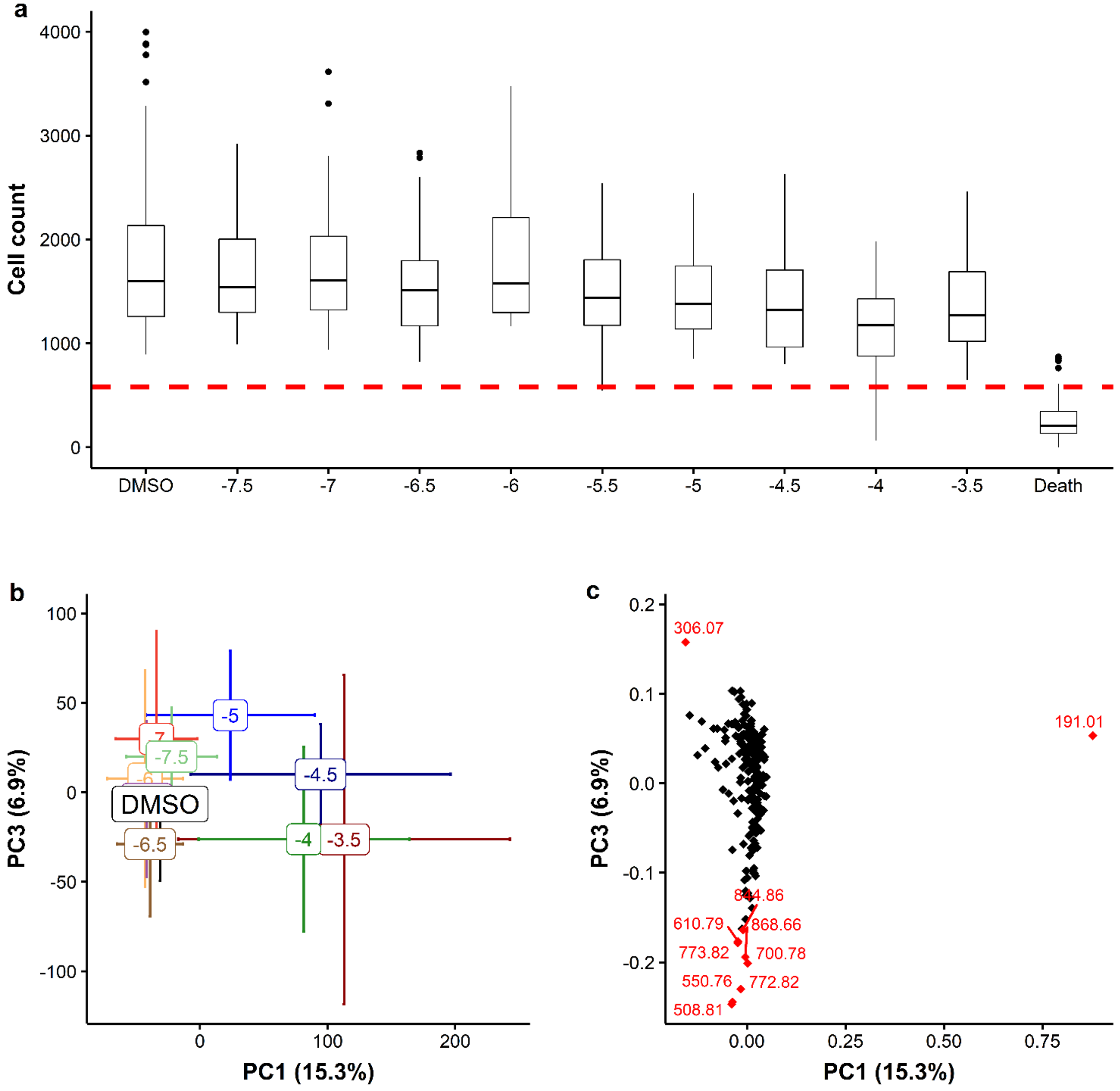

Figure S13. (a) Box plot representation of the cell count in each deferoxamine concentration group and DMSO-controls from the cell imaging data, where the red line indicates the cell phenotype filter applied. PCA scores plot (b) from AMI-MS metabolomics analysis of the HepG2 toxicity study highlighting the metabolic responses of deferoxamine and the corresponding loadings plot (c).

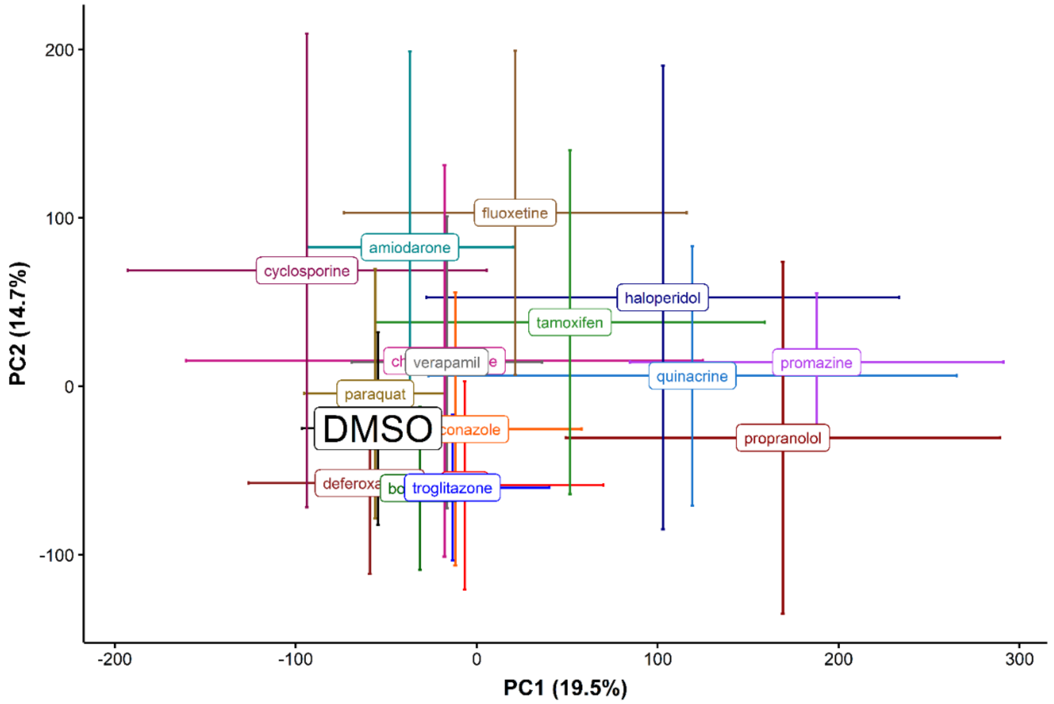

Figure S14. PCA scores plot from AMI-MS analysis of the highest non-cytotoxic concentration of each of the 16 compounds from the HepG2 toxicity study samples; showing clustering based on metabolic perturbations.

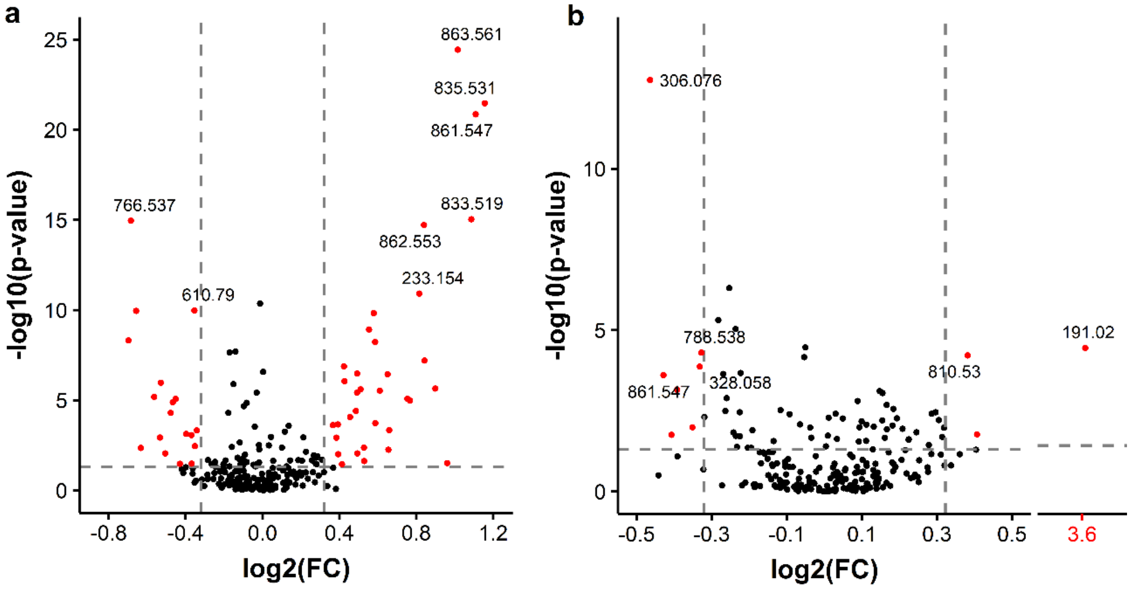

Figure S15. Volcano plots from AMI-MS metabolomics analysis of the HepG2 toxicity study focusing on the tamoxifen (a) and deferoxamine (b) samples. The p-value is derived from applying ANOVA across the concentration range, with the significantly changing features colored red.

References

(1) Roberts, K.; Callis, R.; Ikeda, T.; Paunovic, A.; Simpson, C.; Tang, E.; Turton, N.; Walker, G. Implementation and Challenges of Direct Acoustic Dosing into Cell-Based Assays. *J. Lab. Autom.* **2016**, *21* (1), 76–89.

(2) Lloyd, G. R.; Jankevics, A.; Weber, R. J. M. Struct: An R/Bioconductor-Based Framework for Standardized Metabolomics Data Analysis and Beyond. *Bioinformatics* **2021**, *36* (22–23), 5551–5552.

(3) Sumner, L. W.; Amberg, A.; Barrett, D.; Beale, M. H.; Beger, R.; Daykin, C. A.; W-M Fan, T.; Fiehn, O.; Goodacre, R.; Griffin, J. L.; Hankemeier, T.; Hardy, N.; Harnly, J.; Higashi, R.; Kopka, J.; Lane, A. N.; Lindon, J. C.; Marriott, P.; Nicholls, A. W.; Reily, M. D.; Thaden, J. J.; Viant, M. R. Proposed Minimum Reporting Standards for Chemical Analysis Chemical Analysis Working Group (CAWG) Metabolomics Standards Initiative (MSI) NIH Public Access. *Metabolomics* **2007**, *3* (3), 211–221.

(4) Zhou, W.; Yang, S.; Wang, P. G. Matrix Effects and Application of Matrix Effect Factor. *Bioanalysis* **2017**, *9* (23), 1839–1844.

(5) Viant, M. R.; Ebbels, T. M. D.; Beger, R. D.; Ekman, D. R.; Epps, D. J. T.; Kamp, H.; Leonards, P. E. G.; Loizou, G. D.; MacRae, J. I.; van Ravenzwaay, B.; Rocca-Serra, P.; Salek, R. M.; Walk, T.; Weber, R. J. M. Use Cases, Best Practice and Reporting Standards for Metabolomics in Regulatory Toxicology. *Nat. Commun.* **2019**, *10* (1).

(6) Dieterle, F.; Ross, A.; Schlotterbeck, G.; Senn, H. Probabilistic Quotient Normalization as Robust Method to Account for Dilution of Complex Biological Mixtures. Application in 1 H NMR Metabonomics. *Anal. Chem.* **2006**, *78* (13), 4281–4290.
